# Alkhurma Hemorrhagic Fever in Travelers Returning from Egypt, 2010

**DOI:** 10.3201/eid1612101092

**Published:** 2010-12

**Authors:** Fabrizio Carletti, Concetta Castilletti, Antonino Di Caro, Maria R. Capobianchi, Carla Nisii, Fredy Suter, Marco Rizzi, Alessandra Tebaldi, Antonio Goglio, Cristiana Passerini Tosi, Giuseppe Ippolito

**Affiliations:** Author affiliations: “Lazzaro Spallanzani” National Institute for Infectious Diseases, Rome, Italy (F. Carletti, C. Castilletti, A. Di Caro, M.R. Capobianchi, C. Nisii, G. Ippolito);; “Ospedali Riuniti di Bergamo,” Bergamo, Italy (F. Suter, M. Rizzi, A. Tebaldi, A. Goglio, C.P. Tosi)

**Keywords:** Alkhurma, viral hemorrhagic fever, Flavivirus, zoonoses, Egypt, viruses, dispatch

## Abstract

Two travelers returning to Italy from southern Egypt were hospitalized with a fever of unknown origin. Test results showed infection with Alkhurma virus. The geographic distribution of this virus could be broader than previously thought.

Alkhurma virus (ALKV) is a recently described member of the tick-borne hemorrhagic fever group of the genus *Flavivirus*. It was initially isolated in the late 1990s (*1**,**2*) and is today considered a variant of the Kyasanur Forest disease virus, sharing 89% nt sequence homology (*3**,**4*). This emerging pathogen causes signs and symptoms such as fever, headache, joint pain, muscle pain, vomiting, and thrombocytopenia; severe cases may have hemorrhagic manifestations (epistaxis, ecchymoses, petechiae, hematemesis) and encephalitis, which can result in death (reported case-fatality rate as high as 25%) (*5**–**8*). Camels and sheep are thought to be the natural hosts of ALKV, but whether other mammals are also involved in its life cycle remains unknown. ALKV RNA was recently detected in an *Ornithodoros savignyi* tick collected near Jeddah, Saudi Arabia (*9*); on the Arabian Peninsula, these ticks have been associated with camels and their resting places and can be found where cases of ALKV infection in humans have been reported. These ticks seek multiple hosts, are nocturnal and cryptic, and commonly attack humans and other animals resting under trees (*10*). The hypothesis that mosquitoes could also be vectors has been suggested by 2 studies (*6**,**7*); despite the absence of data to substantiate it, this possibility cannot be excluded.

Evidence suggests that ALKV infects humans either transcutaneously (by contamination of a skin wound with the blood of an infected vertebrate or through the bite of an infected tick) or orally through consumption of unpasteurized contaminated milk. Transmission to humans has been associated with butchering of sheep and camels. No human-to-human transmission has been reported. ALKV is classified in different countries as a BioSafety Level 3 or 4 agent.

ALKV has been detected only in Saudi Arabia, but the closely related Kyasanur Forest disease virus has spread as far as India and the People’s Republic of China (*4*). We describe 2 cases of Alkhurma hemorrhagic fever in 2 travelers who returned to Italy from Egypt in 2010.

## The Cases

The first patient, a 64-year-old man from Italy, spent 1 week (April 25–May 1, 2010) in a touristic village in southern Egypt, near the Sudan border. While visiting a camel and dromedary market in Shalatin on April 29, he was bitten on the foot by an unidentified arthropod (although not formally identified, was described as tick shaped). Soon after, a small, papular lesion developed. During his return flight to Italy, ≈48 hours after the bite, the patient experienced high fever, shaking chills, anorexia, malaise, nausea and vomiting, and blurred vision. During the next 5 days, these signs and symptoms worsened, and the man was admitted to the “Ospedali Riuniti di Bergamo” in northern Italy. His medical history was unremarkable, but he frequently traveled abroad and had been vaccinated against yellow fever in 1998.

Laboratory test results showed leukopenia (2,250 cells/mm^3^), thrombocytopenia (67,000 platelets/mm^3^), and increased liver enzymes (aspartate transaminase 469 U/L, reference 3–46 U/L; alanine transaminase 406 U/L, reference 3–46 U/L). The patient was given acetaminophen, and fever and general malaise progressively decreased over the next 5 days. He was discharged 11 days later, on May 17, in good general condition despite persistence of asthenia.

Acute-phase and convalescent-phase serum samples (collected on May 10 and 27, respectively) were sent to the virology laboratory of the “Lazzaro Spallanzani” National Institute for Infectious Diseases in Rome to be tested for dengue and West Nile viruses. Immunoglobulins (Ig) G and M for both viruses were detected by immunofluorescence of both samples; for each virus, IgG titer was >640 and IgM titer was >20. No evidence of rising antibody titers was found in the convalescent-phase specimen, raising suspicion of cross-reactivity to a previous *Flavivirus* infection or yellow fever vaccination. A genus-specific reverse transcription–PCR selective for the nonstructural protein (NS) 5 gene of flaviviruses (*11*) was positive for the acute-phase and negative for the convalescent-phase samples. Sequence analysis of the amplicon (GenBank accession no. HM629507) showed high similarity with ALKV sequences in GenBank (BLAST [www.ncbi.nlm.nih.gov/blast/Blast.cgi] submission showed 97% identity with AF331718). This unexpected result called for further investigations to confirm the diagnosis of an ALKV infection. Thus, an ALKV-specific nested reverse transcription–PCR selective for a wider region of a different gene (E) was designed by using the following primers: outer forward 5′-TGGAACCCCACACGGGTGACT-3′; outer reverse 5′-ATGCCCACTGTCGGTTGGCG-3′; inner forward 5′-CCCACAGCAATCGAAAAACGGCATC-3′; inner reverse 5′- GCCCACATCACAGGTGACATGACC-3′.

**Figure 1:**
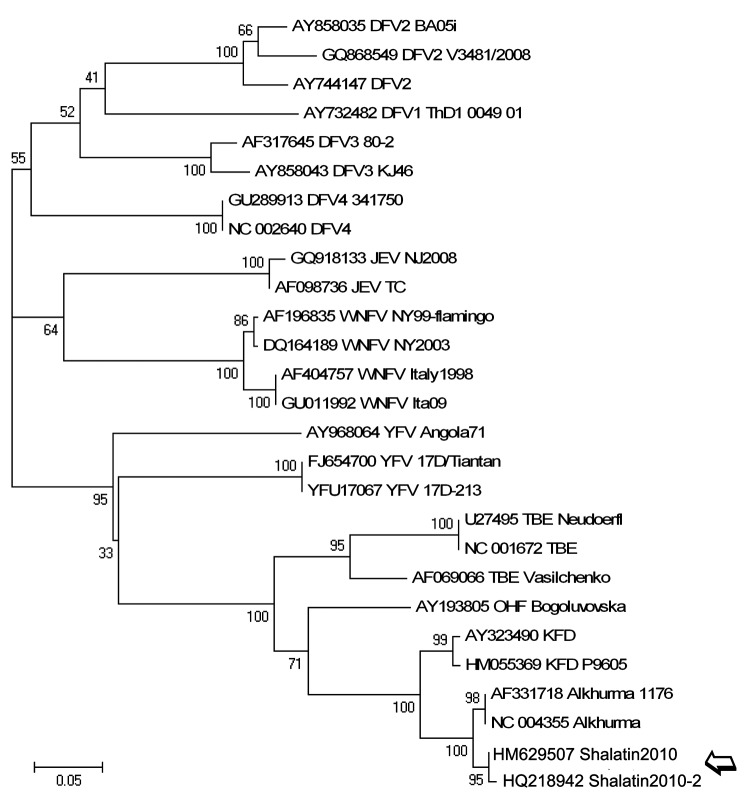
Phylogenetic tree based on sequences of the amplicon produced by the flavivirus nonstructural protein (NS) 5 gene reverse transcription–PCR (amplicon size, 208 bp; position in reference AF331718, nt 9077–9275), performed on the acute-phase serum samples of 2 travelers returning to Italy from Egypt (open arrow) showing relationship with other flaviviruses. Sequences are identified by name and GenBank accession number. Multiple alignment of other flavivirus sequences available in GenBank was generated by use of the ClustalW 1.7 software (www.clustal.org) included in the Bioedit package (www.mbio.ncsu.edu/BioEdit/BioEdit.html). The phylogenetic tree was constructed by nucleotide alignment, the Kimura 2-parameter algorithm, and the neighbor-joining method implemented in MEGA 4.1 software (www.megasoftware.net). The robustness of branching patterns was tested by 1,000 bootstrap pseudo-replications. Scale bar indicates nucleotide substitutions per site. DFV, dengue fever virus; JEV, Japanese encephalitis virus; WNFV, West Nile fever virus; TBEV, tick-borne encephalitis virus; OHFV, Omsk hemorrhagic fever virus; KFDV, Kyasanur Forest disease virus.

**Figure 2:**
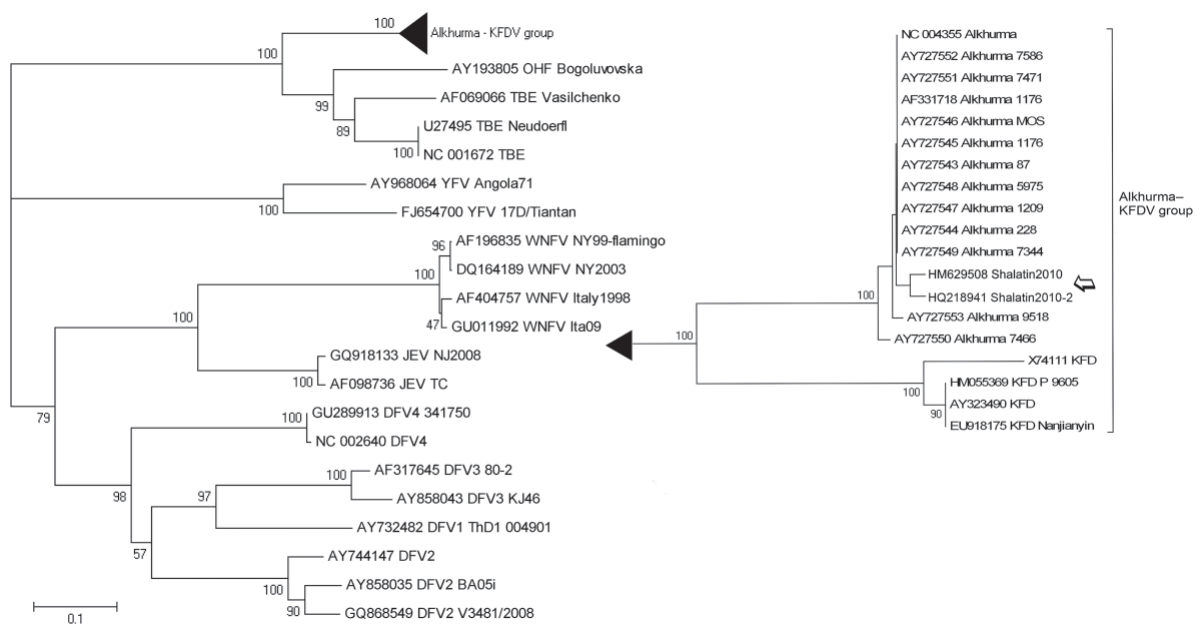
Phylogenetic tree based on the sequences of Alkhurma hemorrhagic fever virus E gene amplicon (amplicon size, 516 bp; position in reference AF331718, nt 1398–1913),obtained from acute-phase serum samples from a patient who had traveled to Egypt (open arrow) with respect to other flaviviruses. Sequences are identified by name and GenBank accession number. The phylogenetic tree was constructed by nucleotide alignment, the Kimura 2-parameter algorithm, and the neighbor-joining method implemented in MEGA 4.1 software (www.megasoftware.net). The robustness of branching patterns was tested by 1,000 bootstrap pseudo-replications. Scale bar indicates nucleotide substitutions per site. DFV, dengue fever virus; JEV, Japanese encephalitis virus; WNFV, West Nile fever virus; TBEV, tick-borne encephalitis virus; OHFV, Omsk hemorrhagic fever virus; KFDV, Kyasanur Forest disease virus. The relevant part of the tree is enlarged at right.

All residual biological samples collected during the patient’s hospital stay were sent to the virology laboratory of the Spallanzani Hospital (Italy’s national reference laboratory for viral hemorrhagic fever viruses, BioSafety Level 4) in compliance with biosafety procedures. The new ALKV PCR result was positive, and the sequence of the amplicon (GenBank accession no. HM629508) showed high homology with ALKV (99% identity with AF331718). The phylogenetic trees based on partial sequences of NS5 (Figure 1) and E (Figure 2) genes confirmed the diagnosis of ALKV infection.

After submitting this article, we detected ALKV infection in a second patient. This patient had traveled to the same area ≈1 month later, visited the same camel market, and was affected by a milder disease. NS5 (HQ218942) and E (HQ218941) gene sequences obtained from this patient have been included in the phylogenetic tree, showing that they cluster together with those from the first patient (Figure 1, Figure 2).

## Conclusions

The 2 patients had traveled to an area of the world where ALKV had not been previously reported. Although viremia was demonstrated 10 days after symptom onset, and we can reliably suppose that it started when fever and chills appeared, the probability of a susceptible vector in Europe is small, and the infection seems not to be transmissible from human to human.

Laboratory diagnosis of this infection is not easy to obtain and requires a specialized laboratory because of antibody cross-reactivity with other members of the family *Flaviviridae* and because of the absence of commercially available serologic tests and reference biologic materials for their development. However, surveillance of travelers returning from areas where highly dangerous infectious diseases are endemic should be improved and should include ALKV. The finding that the distribution of this virus is wider than previously thought and that it includes the African continent is in line with the hypothesis that tick-borne flaviviruses originated in Africa (*12*). The low genetic distance between the Egypt and Saudi Arabia sequences supports the hypothesis of a recent divergence from Kyasanur Forest disease virus, i.e., the closest flavivirus (*5*), and a slow microevolution of ALKV, as for other tick-borne flaviviruses (*13*). The higher genetic divergence in the NS5 gene than in the E gene of ALKV strains confirms previous observations for viruses isolated from human samples after inoculation of suckling mice (*5*) and deserves more detailed evolutionary analysis.

The detection of 2 independent infection events for travelers who visited the same area in a restricted period strongly supports the hypothesis of sustained local ALKV circulation. Further veterinary and entomologic investigations are needed to expand understanding of the geographic distribution of ALKV and to assess the danger for local populations and visitors. It would be advisable to inform travelers about the danger of coming into contact with infected animals in areas where the virus has been reported. Avoidance of or minimization of exposure to infected ticks should be recommended as the most effective prevention measure.
